# Robotic-assisted Esophagectomy vs Video-Assisted Thoracoscopic Esophagectomy (REVATE): study protocol for a randomized controlled trial

**DOI:** 10.1186/s13063-019-3441-1

**Published:** 2019-06-10

**Authors:** Yin-Kai Chao, Zhi-Gang Li, Yu-Wen Wen, Dae-Joon Kim, Seong-Yong Park, Yu-Ling Chang, Pieter C. van der Sluis, Jelle P. Ruurda, Richard van Hillegersberg

**Affiliations:** 1 Division of Thoracic Surgery, Chang Gung Memorial Hospital-Linko, Chang Gung University, Taoyuan, Taiwan; 2 Division of Thoracic Surgery Shanghai Chest Hospital, Shanghai, China; 3 Clinical Informatics and Medical Statistics Research Center Chang Gung University, Taoyuan, Taiwan; 4 Department of Thoracic and Cardiovascular Surgery, Yonsei University College of Medicine, Seoul, Republic of Korea; 5 School of Nursing, Chang Gung University, Taoyuan, Taiwan; 6 Department of Surgery, University Medical Center Utrecht, Utrecht, the Netherlands

**Keywords:** Esophageal cancer, Robotic esophagectomy, Thoracoscopic esophagectomy, Recurrent laryngeal nerve, Lymph node dissection

## Abstract

**Background:**

Radical lymph node dissection (LND) along the left recurrent laryngeal nerve (RLN) is surgically demanding and can be associated with substantial postoperative morbidity. The question of whether robot-assisted esophagectomy (RE) might be superior to video-assisted thoracoscopic esophagectomy (VATE) for performing LND along the RLN in patients with esophageal squamous cell carcinoma (ESCC) remains open.

**Methods/design:**

We will conduct a multicenter, open-label, randomized controlled trial (Robotic-assisted Esophagectomy vs Video-Assisted Thoracoscopic Esophagectomy (REVATE)) enrolling patients with ESCC scheduled to undergo LND along the RLN. Patients will be randomly assigned to either RE or VATE. The primary outcome measure will be the rate of unsuccessful LND along the left RLN, which will be defined as: failure to remove lymph nodes along the left RLN (i.e., no identifiable nodes on pathology reports); or occurrence of permanent (duration > 6 months) left RLN palsy following LND. Secondary outcomes will include the number of successfully removed RLN nodes, postoperative recovery, length of hospital stay, 30-day and 90-day mortality, quality of life, and oncological outcomes.

**Discussion:**

The REVATE study provides an opportunity to explore whether RE could facilitate LND along the left RLN—a complex surgical procedure that, as of now and with the use of VATE, remains difficult to perform and associated with a significant burden of morbidity.

**Trial registration:**

ClinicalTrials.gov, NCT03713749. Registered on 22 October 2018.

**Electronic supplementary material:**

The online version of this article (10.1186/s13063-019-3441-1) contains supplementary material, which is available to authorized users.

## Background

Metastases to nodes located around the recurrent laryngeal nerve (RLN) occur commonly in patients with esophageal squamous cell carcinoma (ESCC), especially when tumors are located in the middle or the upper third of the esophagus [1–4]. Lymph node dissection (LND) along the RLN is considered beneficial in patients with esophageal cancer because it can result in more accurate disease staging as well as improved local control rates. However, RLN LND is surgically demanding and is frequently complicated by RLN palsy (occurrence rate 20–80%)—which is especially common on the left side [5, 6]. Injury of the RLN induces a paresis or palsy of the vocal cords—which can in turn increase the rates of postoperative pulmonary complications and severely impair quality of life [7–9].

Owing to an improved magnification, the significant advances made in video-assisted thoracoscopic esophagectomy (VATE) over the last decades have led to significant improvements in surgical results [10]. Compared with open surgery, experienced surgical teams may obtain a significant reduction of postoperative complications through the use of VATE [10–12]. Unfortunately, RLN LND remains challenging even in the VATE era—especially on the left side—mainly because of major technical barriers (e.g., limited surgical space, rigidity of the instrumentation, two-dimensional vision) [13]. Importantly, a previous study has shown that VATE is unable to reduce the rates of postoperative RLN palsy following RLN LND [14].

Currently, robotic surgery with three-dimensional stereoscopic 10× magnified visualization systems and flexible wrist mechanisms is a leading technology to improve the accuracy of dissections performed in limited anatomical spaces. Although interest in robot-assisted esophagectomy (RE) is growing [15–17], there are limited studies comparing the robotic approach with VATE [18–20]. Specifically, the question of whether RE might be superior to VATE for performing LND along the RLN in patients with ESCC remains open.

The REVATE study aims to assess the efficacy and safety of RE to perform RLN LND in patients with ESCC. The primary hypothesis is that—compared with VATE (REVATE control group)—the use of RE (REVATE treatment group) can reduce the rate of unsuccessful LND along the left RLN, which will be defined as: failure to remove lymph nodes along the left RLN; or occurrence of permanent left RLN palsy following LND. Secondary objectives are to evaluate the impact of RE on the following variables: number of successfully removed RLN nodes, postoperative recovery, length of hospital stay, 30-day and 90-day mortality, quality of life, and oncological outcomes.

## Methods/design

### Design and setting

**Fig. 1:**
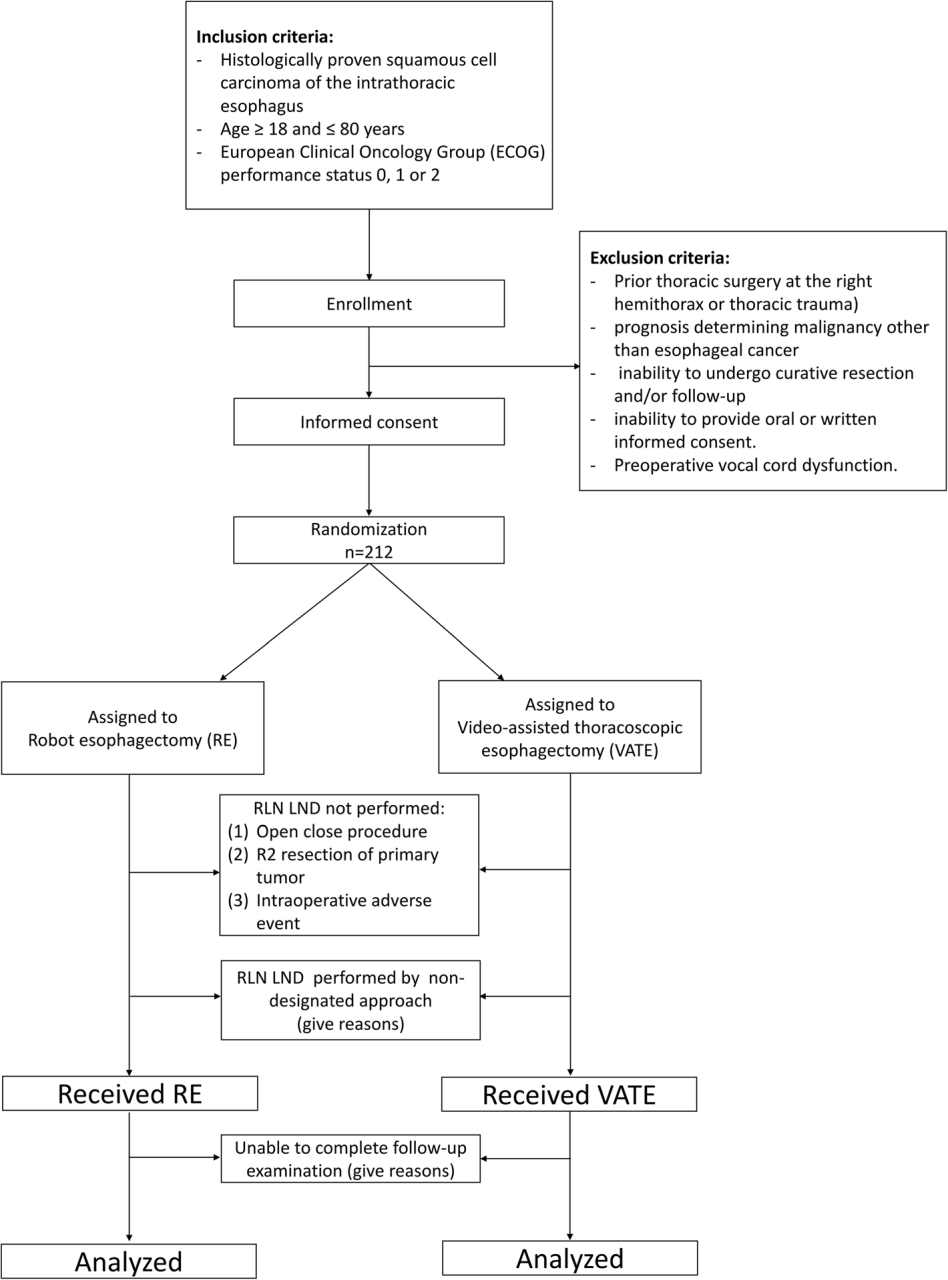
Robotic-assisted Esophagectomy vs Video-Assisted Thoracoscopic Esophagectomy (REVATE) study flowchart. LND lymph node dissection, RLN recurrent laryngeal nerve

A study flowchart is shown in Fig. 1. This is an investigator-initiated, investigator-driven multicenter, open-label, randomized controlled trial (termed REVATE) enrolling patients with ESCC scheduled to undergo LND along the RLN. Patients will be randomly assigned (1:1 ratio) to undergo minimally invasive esophagectomy (MIE) with either RE or VATE. All participating surgeons will be required to have competence with both techniques. The study will take place in two high-volume surgical centers located in Taiwan and China, and will be conducted with adherence to the principles of the World Medical Association’s Declaration of Helsinki.

### Study population: inclusion and exclusion criteria

Our study population will consist of adult patients with ESCC requiring MIE. Inclusion criteria will be as follows: age between 18 and 80 years; histology-proven primary intrathoracic ESCC; scheduled treatment with MIE (McKeown procedure) and bilateral RLN LND; European Clinical Oncology Group performance status 0–1; presence of surgically resectable disease (cT1−4a, N0–3, M0) defined according to the *American Joint Committee on Cancer (AJCC) Staging Manual*, eighth edition [21]; normal bilateral vocal cord function confirmed by preoperative laryngoscopic examination; and willingness to provide written informed consent. If the candidates meet any of the following criteria, they will not be eligible for the study: history of previous major thoracic surgery that renders MIE unfeasible; malignancies different from ESCC; and inability to undergo curative resection and/or to comply with the follow-up schedule.

### Timeline of inclusion and randomization

Patients who do not require preoperative neoadjuvant chemoradiotherapy (nCRT) will be informed about potential inclusion in the first 3 weeks of the study before the scheduled operation. When nCRT will be necessary, candidates will be approached only upon completion of the nCRT course.

### Randomization

A computerized randomization tool will be used to randomly allocate patients to either RE or VATE (1:1 ratio). In order to minimize the potential confounding effect of local treatment variables (e.g., postoperative care), patients will be stratified in a 1:1 fashion according to the enrolling hospital. A permuted-block randomization with varying block size will be implemented. Allocation and block size will be concealed from all investigators. Blinding of surgeons is unfeasible because of the obvious technical differences between RE and VATE. However, the occurrence of the study endpoints will be determined from medical records by an independent assessor who will be blinded to the surgical technique used for each case. Patients will have the option to withdraw from the study at any time.

### Surgical approach

Regardless of the treatment arm (i.e., RE versus VATE), all patients will undergo a total two-field lymphadenectomy according to the consensus proposed in 1994 by the International Society of Disease of Esophagus (ISDE) [22]. The operating surgeon will be exempted from performing RLN LND in the presence of at least one of the following conditions: low surgical curability as evidenced by tumor ingrowth into adjacent organs (i.e., T4b status) or unexpected detection of distant metastases (i.e., M1 status) during surgery; and/or occurrence of previously unforeseen perioperative adverse events and/or findings which will require discontinuation of surgery (all of these events will be detailed on an individual basis). Other surgical variables—including the use of laparotomy versus laparoscopy, the technique used for anastomosis, and the route of reconstruction—are not expected to influence the results of RLN LND and will be left to the surgeon’s discretion.

### Definition of RLN lymph nodes

The location of bilateral RLN lymph nodes will be defined according to the criteria proposed by the Japan Esophageal Society (JES) [23]. Right (station 106recR) and left (station 106recL) RLN lymph nodes correspond, respectively, to stations termed 2R and 2L in the *AJCC Staging Manual*, eighth edition (Table 1).

**Table 1:** Comparisons of the nomenclature and grouping of mediastinal lymph nodes as proposed by the AJCC/UICC and JES systems for patients with esophageal cancer

AJCC/UICC system	JES system
Station 2R: upper right paratracheal lymph nodes	Station 106recR: right recurrent laryngeal nerve nodes
Station 2L: upper left paratracheal lymph nodes	Station 106recL: left recurrent laryngeal nerve nodes
Station 8U: upper thoracic paraesophageal lymph nodes	Station 105: upper thoracic paraesophageal lymph nodes
Station 4R: right lower paratracheal lymph nodes	Station 106pre: pretracheal lymph nodes
	Station 106tbR: right tracheobronchial lymph nodes
Station 4L: left lower paratracheal lymph nodes	Station 106tbL: left tracheobronchial lymph nodes
N/A	Station 113: ligamentum arteriosum lymph nodes
N/A	Station 114: anterior mediastinal lymph nodes
Station 7: subcarinal lymph nodes	Station 107: subcarinal lymph nodes

*AJCC* American Joint Committee of Cancer, *UICC* Union for International Cancer Control, *JES* Japan Esophageal Society, *N/A* not available

### Data collection and management

At inclusion, an unequivocal identification code will be generated for each participant, with its access being restricted to the principal investigator (PI) and the study coordinators. A good clinical practice (GCP)-compliant digital case record form (CRF) will be used for data collection, for which the study coordinators and/or research nurses will be in charge. Nonelectronic data will be secured in locked cabinets located at data coordinating centers, being accessible to the PI, as well as research nurses and physicians. Each participating center will be allowed to request information from the principal database, but direct access will be permitted only for locally generated data. Upon termination of the study, data access requests may be forwarded to the PI. The completed CRFs will be cross-checked with the original sources to ensure that data regarding the primary and secondary outcome measures will be accurate and reliable. All clinical records will be stored anonymously to protect privacy and all investigators will adhere to local confidentiality regulations.

### Primary outcome measure

The primary outcome measure will be the rate of unsuccessful LND along the left RLN, which will be defined as: failure to remove lymph nodes along the left RLN (i.e., no identifiable lymph nodes confirmed by pathology reports); or occurrence of permanent (duration > 6 months) left RLN palsy following LND. Regardless of the presence of hoarseness, vocal cord function will be assessed by an experienced otolaryngologist using a flexible laryngoscope within 1 week of surgery. RLN palsy will be classified according to the following variables: site (unilateral versus bilateral); duration (temporary (i.e., recovering within 6 months) versus permanent (i.e., not recovering within 6 months)); and type of treatment required (type I, no therapy required; type II, injury requiring an elective surgical procedure; type III, injury requiring an urgent surgical procedure) [24]. All patients diagnosed with postoperative RLN palsy will undergo additional laryngoscopic examinations at 1, 3, and 6 postoperative months to confirm the occurrence of permanent RLN palsy (defined as its persistence at 6 months after surgery).

### Secondary endpoints

Secondary endpoints will include: the number of nodes removed along the right and left RLN; the incidence of pneumonia, defined according to the Revised Uniform Pneumonia Score which includes temperature, leukocyte count, and pulmonary radiography (Table 2) [25]; the incidence of other postoperative complications (e.g., anastomotic leakage, chylothorax), defined according to the Esophagectomy Complications Consensus Group system; the total percentage of surgery-related major postoperative complications (i.e., grade IIIa or higher according to the Clavien–Dindo criteria) [26]; in-hospital, 30-day, and 90-day mortality rates, defined as any death occurring during the same hospitalization and within 30 or 90 days after surgery, respectively; R0 resection rates, defined as microscopically negative proximal/distal and circumferential margins; surgery-related parameters (including thoracic, abdominal, and total surgical time (expressed in minutes); unexpected events and complications occurring during surgery (e.g., massive hemorrhage, perforation of other organs); blood loss during surgery (expressed in milliliters per phase); and number of patients requiring conversion to thoracotomy and related reasons); postoperative recovery parameters (including length of mechanical ventilator use after surgery (expressed in minutes), length of ICU stay (expressed in hours), length of hospital stay (expressed in days), and need for reintubation or readmission to the ICU); quality of life and psychometric measures (including the EORTC QLQ-C30, OES-18, and Hospital Anxiety and Depression Scale (HADS) questionnaires, administered at the following time points: 5 days before surgery; 4 weeks, 3 months, and 6 months after surgery; and on a yearly basis up to 5 years thereafter); and oncological outcomes (including 2-year, 3-year, and 5-year disease-free and overall survival rates).

**Table 2:** Revised Uniform Pneumonia Score

Diagnostic criterion	Revised Uniform Pneumonia Score	
	Range	Score
Temperature (°C)	≥ 36.1 and ≤ 38.4	0
	≤ 36.0 and ≥ 38.5	1
Leukocyte count (× 10^9^/L)	≥ 4.0 and ≤ 11.0	0
	< 4.0 or > 11.0	1
Pulmonary radiography	No infiltrate	0
	Diffused (or patchy) infiltrate	1
	Well-circumscribed infiltrate	2

A sum score of 2 points or higher, in which at least 1 point is assigned because of infiltrative findings on pulmonary radiography, indicates the presence of pneumonia

### Quality control

In order to avoid a learning curve bias, only surgeons with a proven track of at least 50 previously performed RE and VATE procedures will be involved in the trial. Every operation performed within the trial will be video-recorded. All of the events of interest (related to the study outcomes) and surgical videos will be regularly reviewed by a local independent committee consisting of surgeons with proven expertise in MIE and involved in the conduct of the trial. All disagreements will be handled by a senior member of the Upper GI International Robotic Association (http://ugira.org/)—whose decision will be final.

### Follow-up schedule

**Fig. 2:**
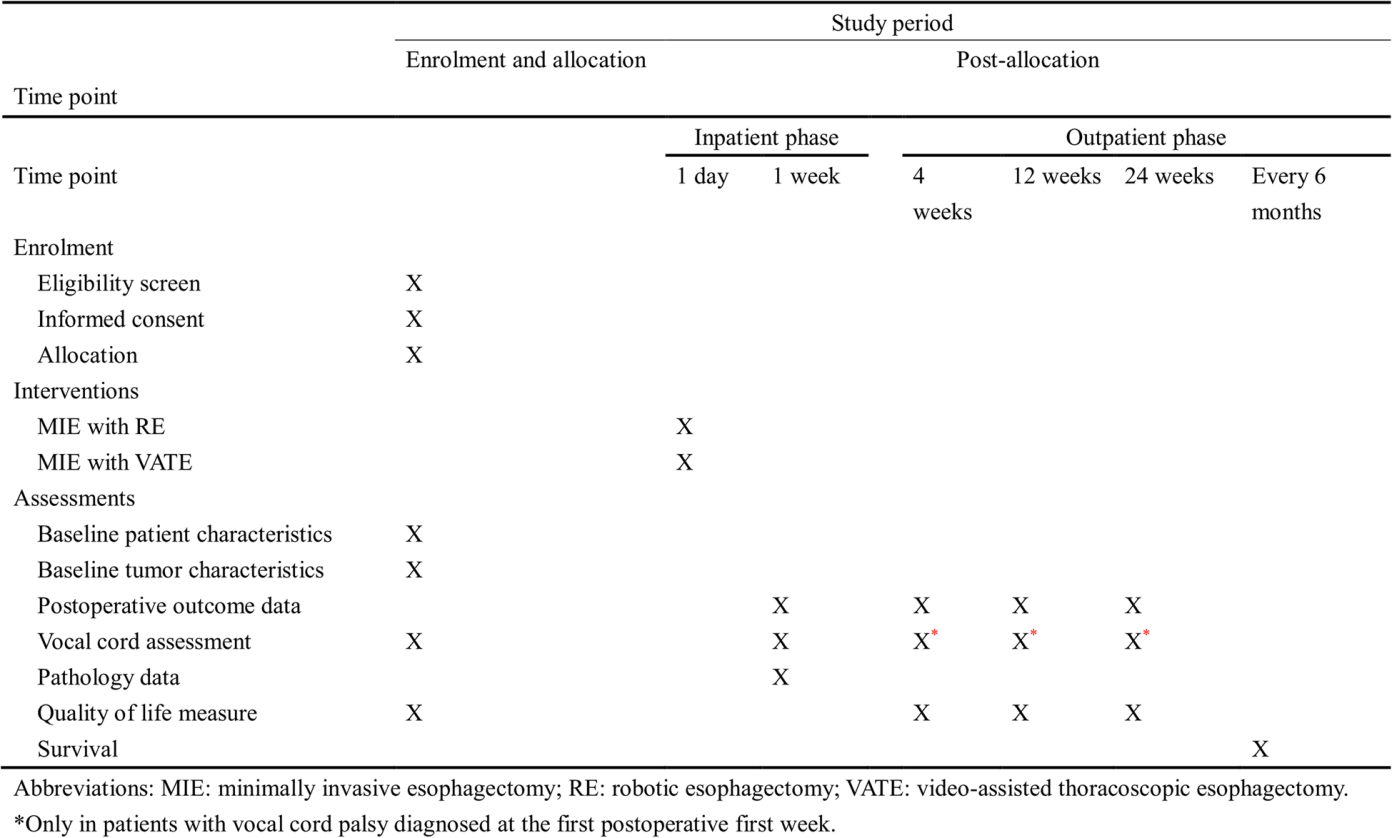
Standard Protocol Items: Recommendations for Interventional Trials (SPIRIT) schedule of patient enrolment, interventions, and assessments

Randomization will be considered the start of the study for each participant. All patients will be followed up according to the schedule shown in Fig. 2. The primary study endpoint will be assessed at 1 week (in the entire study cohort) and 6 months (in the subgroup of patients with confirmed postoperative left side RLN palsy at 1 postsurgical week). Data on postoperative complications, readmissions, and deaths occurring within 30 and 90 postoperative days will be collected. The study visits are planned at the following time points: 2 weeks before surgery; 4 weeks, 3 months, and 6 months after surgery; and every 6 months thereafter (until 5 postoperative years).

### Recruitment and trial timeline

The study started on November 12, 2018, with a projected 3-year inclusion period. Analysis of short-term results and long-term oncological outcomes will be conducted at 6 months and 5 years after discharge of the last randomized patient, respectively. The expected total duration of the trial is 8 years, including prearrangement and data analysis. The investigators will release interim reports at the end of each preplanned follow-up period.

### Sample size calculation

Based on a preliminary study conducted by our group, the projected rates of successful left RLN LND (according to the definition proposed in the current trial) are 70–75% and 85–90% for VATE and RE, respectively [20]. The purpose of our study is to demonstrate that the rate of successful left RLN LND will be at least 15% higher for RE compared with VATE (based on a conservative 75% rate for VATE). Because our null hypothesis is that the difference between the two arms is ≤15%, we will adopt one-sided testing. In a one-sided test, the “extreme” portion of distribution is decided beforehand as meaning “sufficiently large” (i.e., 15% in our case) [20]. Assuming an alpha error of 0.05 and an 80% power using one-sided two-sample comparisons of proportions under a balanced trial design, a total of 95 patients per treatment arm will be required. Assuming a dropout rate of 10% in the entire study cohort, we plan to enroll a total of 212 patients (i.e., 106 in each arm).

### Statistical analysis

A formal statistical analysis plan will be prepared by both an independent statistician and the principle investigator ahead of the final data collection (both blinded to treatment allocation and the study results). The final study findings will be released by the study statistician. Data analysis will be performed in patients who will receive RLN LND according to the procedure assigned at randomization and who will have complete follow-up data on nerve palsy recovery status at the 6-month assessment (for confirmation of permanent nerve palsy). A small percentage of patients (estimated 5% maximum) are expected not to undergo the procedure assigned at randomization either because of self-withdraw from the study or because of occurrence of unexpected intraoperative findings (as described in the “Surgical approach” section). Another 5% of patients may potentially be lost to follow-up, resulting in a maximum expected total dropout rate of 10%. We will perform both an in-depth analysis of the reasons for dropout and a comparison of dropouts and completers. The primary outcome will be analyzed using a two-sample proportion test (single-sided). Categorical secondary outcomes will be expressed as frequencies and compared with the Fisher’s exact test. Continuous secondary outcomes will be given as means ± standard deviations (for normally distributed variables) or medians and interquartile ranges (for skewed parameters). Comparisons will be performed using Student’s *t* test and the nonparametric Mann–Whitney *U* test, respectively. The Cochran–Mantel–Haenszel test will be used to examine the differences in categorical outcomes between the two treatment groups after adjusting for study center and use of nCRT. All calculations will be conducted with SAS (version 9.3; SAS Institute Inc., Cary, NC, USA) and SPSS (version 20.0; SPSS Inc., Chicago, IL, USA) statistical packages. *P* < 0.05 will be considered statistically significant.

### Interim analysis

An interim analysis will be conducted. We will use the Peto approach (i.e., *P* < 0.001) as the stopping rule for the primary efficacy endpoint (i.e., better outcomes for the RE group). The trial will not be stopped for futility (i.e., lack of differences between the VATE and RE groups) because the robot-assisted minimally invasive approach is increasingly being used by a number of different surgical centers worldwide and all of the endpoints of this randomized trial are expected to be of interest to healthcare professionals involved in this surgical procedure. As advised by the Central Committee on Research involving Human Subjects, no formal stopping rule for harm will be adopted. Upon recruitment of 50 patients, individualized patient description charts (including safety parameters) will be presented to the Data Safety Monitoring Committee (DSMC). The procedure will be repeated every 50 patients. Cases will be discussed by the DSMC in a plenary or telephone conference in the presence of the study coordinator and the PI. In the case of worse outcomes occurring in the RE group, the DSMC will inform the trial research group. The potential harm to each patient will be discussed by the DSMC and the trial research group in a plenary session aimed at determining whether an association exists between the observed adverse events and the use of RE. When a consensus will be reached, the Institutional Review Board will be informed accordingly.

## Discussion

**Fig. 3:**
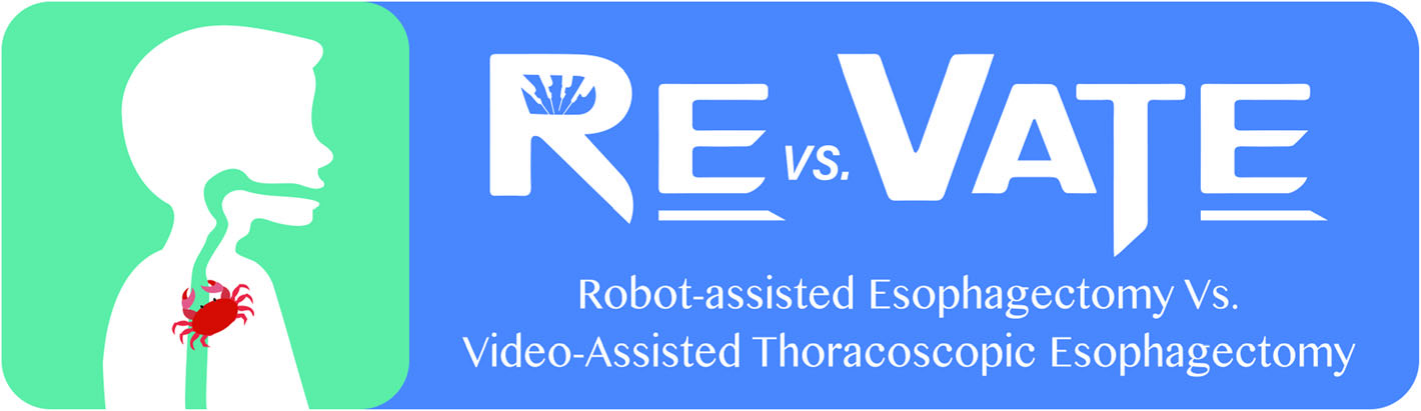
Robotic-assisted Esophagectomy vs Video-Assisted Thoracoscopic Esophagectomy (REVATE) trial logo

The key prerequisite for reducing the burden of RLN palsy following RLN LND is a reliable identification of the anatomical course of the left RLN—which is unfortunately difficult to achieve. Blunt dissection and removal of lymph nodes located around the RLN may cause nerve contusions and heat injuries that can in turn result in postoperative palsy. In this scenario, RE holds great promise for reducing the likelihood of RLN damage owing to its inherent advantages, including the possibility to obtain a precise hemostasis and the capacity of performing multiaxial movements in a tension-free manner. Interest in RE is mounting, but only a paucity of studies have directly compared its results with those of VATE. Moreover, published studies have been limited by their retrospective design and lack of randomization. Importantly, the use of RE has been restricted to patients who expressed their willingness to undergo a partially insured robot-assisted operation (potentially introducing a selection bias related to the high socioeconomic status of patients for whom RE was affordable). A randomized allocation to either RE or VATE (as proposed in our current protocol) is expected to minimize the confounding impact of this variable. To our knowledge, there are no randomized trials of RE versus VATE, although a published randomized study has compared RE with open thoracotomy [27]. We believe that the REVATE study (Fig. 3) provides an opportunity to explore whether RE could facilitate LND along the left RLN—a complex surgical procedure that, as of now and with the use of VATE, remains difficult to perform and associated with a significant burden of morbidity in ESCC patients. If positive, our results will pave the way for a more widespread use of RE in ESCC, as well as for further cost-effectiveness comparative analyses of the two techniques.

## Trial status

Ethics approval was granted from each participating institution before starting enrollment and consent will be obtained for each patient following local regulations. The protocol (20,180,828 version 2) has been prepared and reported in accordance with the Standard Protocol Items: Recommendations for Interventional Trials. The study was initiated on November 12, 2018 as originally planned, with a projected 3-year inclusion period. Analysis of short-term results and long-term oncological outcomes will begin at 6 months and 5 years after discharge of the last randomized patient, respectively.

## Additional file

Additional file 1: SPIRIT 2013 Checklist: Recommended items to address in a clinical trial protocol and related documents (DOC 123 kb)

## Data Availability

Raw data will be available from the corresponding author upon reasonable request. Transfers of clinical data will require approval from the Institutional Review Board.

## Abbreviations

CRF: Case record form
ESCC: Esophageal squamous cell carcinoma
GCP: Good clinical practice
HADS: Hospital Anxiety and Depression Scale
LND: Lymph node dissection
MIE: Minimal invasive esophagectomy
nCRT: Neoadjuvant chemoradiotherapy
RE: Robot-assisted esophagectomy
RLN: Recurrent laryngeal nerve
VATE: Video-assisted thoracoscopic esophagectomy
